# Daily milk consumption and all-cause mortality, coronary heart disease and stroke: a systematic review and meta-analysis of observational cohort studies

**DOI:** 10.1186/s12889-016-3889-9

**Published:** 2016-12-08

**Authors:** Patrick Mullie, Cécile Pizot, Philippe Autier

**Affiliations:** 1 International Prevention Research Institute, Espace Européen, Building G, Allée Claude Debussy, Lyon Ouest, Ecully, 69130 France; 2 Vrije Universiteit Brussel, Elsene, Belgium; 3 University of Strathclyde Institute of Global Public Health at iPRI, International Prevention Research Institute, Ecully, France

## Abstract

**Background:**

Observational studies and meta-analyses relating milk consumption by adults to all-cause mortality, coronary heart disease and stroke have obtained contradictory results. Some studies found a protective effect of milk consumption, whilst other found an increased risk.

**Methods:**

We performed a systematic literature search until June 2015 on prospective studies that looked at milk consumption, all-cause mortality, coronary heart disease and stroke. Random-effect meta-analyses were performed with dose-response.

**Results:**

Twenty-one studies involving 19 cohorts were included in this meta-analysis, 11 on all-cause mortality, 9 on coronary heart disease, and 10 on stroke. Milk intake ranged from 0 to 850 mL/d. The summary relative risk (SRR) for 200 mL/d milk consumption was 1.01 (95% CI: 0.96–1.06) for all-cause mortality, 1.01 (95% CI: 0.98–1.05) for fatal and non fatal coronary heart disease, and 0.91 (95% CI: 0.82–1.02) for fatal and non fatal stroke. Stratified analyses by age, Body Mass Index, total energy intake and physical acitivity did not alter the SRR estimates. The possibility of publication bias was found for all cause mortality and for stroke, indicating a gap in data that could have suggested a higher risk of these conditions with increased milk consumption.

**Conclusions:**

We found no evidence for a decreased or increased risk of all-cause mortality, coronary heart disease, and stroke associated with adult milk consumption. However, the possibility cannot be dismissed that risks associated with milk consumption could be underestimated because of publication bias.

**Electronic supplementary material:**

The online version of this article (doi:10.1186/s12889-016-3889-9) contains supplementary material, which is available to authorized users.

## Background

Milk consumption is recommended by many nutritional guidelines for meeting daily requirements for calcium, animal proteins and vitamin B12 intake. In the United-States, the national dietary guidelines recommend that adults should drink three cups or 732 mL/d of milk [1]. Such level of consumption is, however, rarely observed. According to the Canadian Dairy Information Centre [2], the mean per capita milk consumption in 2014 in the United States was 196 mL/d, in Europe 171 mL/d, with great heterogeneity in consumption ranging from 236 mL/d in Sweden, 171 mL/d in Italy to less than 60 mL/d in Bulgaria.

Despite of its positive image, some nutritionists question the place of cow’s milk in human nutrition in view of its high energetic content, i.e., 83 kcal and 149 kcal for one cup non fat milk and whole milk, respectively [3]. Moreover, whole milk is rich in saturated and transfatty acids [4].

Despite the fat content of whole milk, a meta-analysis of prospective studies published in 2011 found that the consumption of 200 mL/d of milk was associated with a statistically significant 6% reduction in the risk of cardiovascular disease (summary relative risk [SRR] 0.94; 95% CI: 0.89–0.99) [5]. The SRR for all-cause mortality was 0.99 (95% CI: 0.95–1.03). In contrast, a large Swedish prospective study published in 2014 found that 200 mL/d of milk intake was associated with a relative risk for cardiovascular mortality of 1.15 (95% CI: 1.12–1.19) in women and 1.05 (95% CI: 1.03–1.07) in men [6]. Moreover the most adjusted relative risk for all-cause mortality was 1.15 (95% CI: 1.13–1.17) in women and 1.03 (95% CI: 1.01–1.04) in men, for an average milk consumption of 200 mL/d [6]. This heterogeneity in results is less present in meta-analyses for dairy products in general. The most recent meta-analysis found an overall SRR of 0.88 (95% CI: 0.81–0.96) with an I^2^ of 30% for dairy consumption and cardiovascular diseases [7].

In view of these contradictory results observed for milk consumption only, and knowing that since 2010 eight prospective studies have been newly published, we performed an updated meta-analysis in order to clarify reasons underlying these contradicting results, limiting the analysis to milk consumption, without inclusion of dairy products.

## Method

### Literature search and study selection

A systematic search and quantitative analysis was planned, conducted and reported following PRISMA guidelines regarding meta-analysis of observational studies [8]. Published reports until 30/06/2015 were retrieved from Embase, Web of Science, and PubMed using MesH index terms “milk” OR “dairy”, combined with “mortality”, OR “coronary heart disease”, OR “stroke”. Hand searches in reference lists of retrieved articles and of other systematic reviews were also performed. Studies were eligible for inclusion in our meta-analysis if they had a prospective design, and if results related to milk intake only were reported, irrespective of the type of milk and not in association with any other dairy product. Because we were willing to estimate a dose-response relationship, milk intake had to be reported in three categories or more.

Titles were screened to exclude studies in animals, studies in children and in sick people. In a second step, abstracts of the articles were screened for the inclusion criteria. For selected articles, full texts were retrieved and fully read by at least two co-authors.

### Data extraction

Milk intake data as well as most adjusted relative risks with 95% confidence interval were extracted from selected articles. The article selection and data extraction processes were done by two independent reviewers. If for the same study, several articles had been published on the same exposure and outcome, only the most recent publication was retained. Data extraction also involved key study characteristics including the stratified analyses that had been done and the possible confounders that were adjusted for. The selected outcomes were all-cause mortality, fatal and non fatal coronary heart disease and fatal and non fatal stroke.

Some studies reported results on cardiovascular diseases [6, 9–14]. However, a careful reading of articles highlighted that conditions encapsulated by the term “cardiovascular diseases” were quite variable across studies, from cardiovascular mortality [10] to the full range of ICD-10 codes corresponding to cardiovascular diseases [6]. We therefore decided to not perform a meta-analysis on outcomes labelled as fatal and non fatal cardiovascular disease occurrence.

For Mann et al. [15], the death rate ratios were converted to risk ratios, and for Abbott et al. [16], cumulative incidences from a figure were converted in risk ratios and supposed expressed for quartiles of milk intake. For Larsson et al. [17], Goldbohm et al. [13] and Hu et al. [18], fixed-effect meta-analyses were done using risk estimates for whole milk and low-fat milk in order to obtain a single relative risk for milk. For Goldbohm et al. [13], a second fixed-effect meta-analysis was done using sex-specific risk estimates in order to obtain a single relative risk for both sexes. From this last publication, risk estimates prevalent cases for non fermented milk were extracted, because risk estimates for total milk included also fermented milk. The articles by Appleby et al. [19], Hu et al. [18], Iso et al. [20], and Al Delaimy et al. [21] did not report results on milk intake and mortality, coronary heart disease and stroke. However, relevant data from these studies were reported in Soedamah-Muthu et al. [5].

### Statistical analysis

Milk consumption was converted from servings or other units into mL/d by using standard conversions from the Food Standards Agency [22]. One serving or glass of milk was estimated to equate to 200 mL on average [22].

The dose-response meta-analysis was carried out in programming language R (*version 2.13.1, GNU General Public License, 2011*). For dose-response analysis, a first linear model was fitted, within each study, to estimate the relative risk per one unit of drink increase. When the number of subjects at each category of exposure was reported, the model was fitted according to the method proposed by Greenland et al. [23] which provides the natural logarithm of relative risk, and an estimator of its standard error, taking into account the fact that the estimates for separate categories depend on the same reference group [23].

All statistical tests were two-sided with significance level at 0.05. Forest plots were drawn for the relation between milk and all-cause mortality, coronary heart disease, and stroke.

Between-study heterogeneity across studies included in the random-effect meta-analysis was evaluated by *I* ^*2*^, which represents the percentage of total variation across studies that is attributable to heterogeneity rather than to chance [24]. To investigate sources of heterogeneity, separate analyses were performed for studies adjusted for age, Body Mass Index (BMI), total energy intake and physical activity.

Statistically significant results are more likely to be easily and quickly published in international peer-reviewed journals. Null or non-significant results are harder to publish. This has to be taken into account in meta-analyses because this may introduce publication bias. Publication bias was assessed using the Macaskill test [25], the Egger test [26], the Begg test [27] and funnel plots for all-cause mortality, coronary heart disease and stroke. Sensitivity analyses were carried out to evaluate the influence of individual studies with major deviating results (i.e., Michaëlsson et al. [6] and Hu et al. [18]) and for studies with data extracted from figures (i.e., Abbott et al. [16]).

## Results

**Fig. 1:**
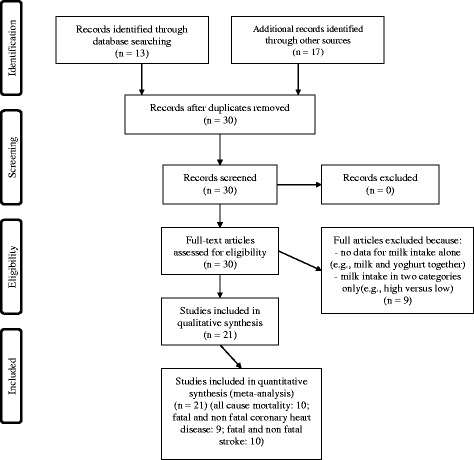
Prisma flow chart of selections of studies included in the meta-analyses relating daily milk consumption to all-cause mortality, coronary heart disease and stroke

We included 21 studies in our meta-analysis, involving 19 cohorts (Fig. 1 and Additional file 1: Table S1) [6, 9, 10, 12–21, 28–35]. We included one study published in 1984 [16] that was not included in the meta-analysis of Soedamah-Muthu et al. [5] We excluded five studies selected by Soedamah-Muthu et al. [5] because of absence of data specific to milk consumption [36, 37] or data were about dairy products and not about milk alone [11, 38, 39]. Engberink et al. [37], that was included in the meta-analysis of Soedamah-Muthu et al. [5], was a poster presentation on the Rotterdam Study that did not report risk estimates for milk consumption. Results of the Rotterdam study on milk consumption and stroke or coronary heart disease were published by Praagman et al. [33], which we included in this meta-analysis. We exluded Elwood et al. [40] for stroke and ischemic heart disease because it was a subsample of Elwood et al. [9]; Mann et al. [15] for coronary heart disease because it was a duplicate of Appleby et al. [19]; Whiteman et al. [41] for all-cause mortality and ischemic heart disease because no quantity of milk consumption was reported for these outcomes. We could not include Avalos et al. [42] and Snowdon et al. [43] in the meta-analysis because outcomes were reported in two categories, which precludes a dose-response analysis.

Five cohorts were conducted in the United States, two in Asia, 11 in Europe, and one in Australia (Additional file 1: Table S1). The age range of study participants at cohort inception was 34 to 74 years. Mean (SD) cohort follow-up was 16 (5) years. We categorised the cohorts according to outcomes (Additional file 1: Table S1): 11 cohorts including 281,788 subjects reported on 63,545 all-cause deaths, nine cohorts including 403,776 sujects reported on 37,049 cases of fatal and non fatal coronary heart disease, and 10 cohorts including 564,717 subjects reported on 39,352 cases of fatal and non fatal cases of stroke. The median milk intake ranged from 0 to 850 mL/d. Adjustment for age, smoking, physical activity, total energy intake and BMI was done in nine studies [6, 12–14, 17, 18, 21, 30, 35].

**Fig. 2:**
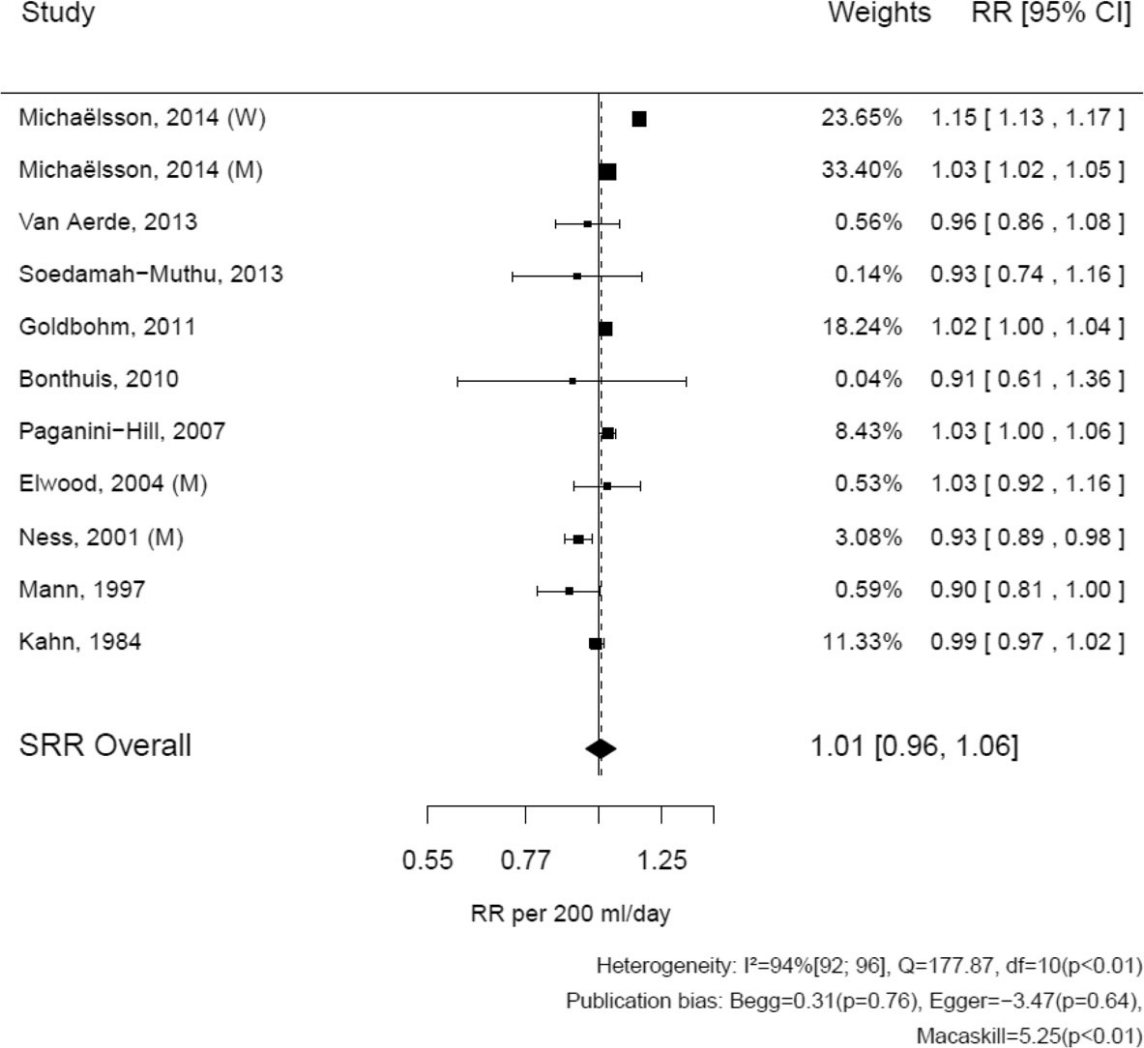
Relation between milk (per 200 mL/d) and all-cause mortality: dose-response meta-analyses of eleven prospective cohorts. Author names, year of publication, and the size of the association per study; the *horizontal lines* indicate 95% confidence intervals. The last two columns contain the actual estimated relative risks and 95% confidence intervals. On the x axis, a line is plotted through the relative risk = 1. The diamond at the *bottom* indicates the summary relative risk with 95% confidence interval. Tests for heterogeneity and publication bias are mentioned at the *bottom*

For all cause deaths, the risks reported by studies ranged from 0.90 (95% CI: 0.81–1.00) to 1.15 (95% CI: 1.13–1.17) (Fig. 2) [6, 15]. The SRR for all-cause deaths associated with the consumption of 200 mL/d of milk was of 1.01 (95% CI: 0.96–1.06). The heterogeneity of results across studies was considerable (I^2^ = 94%, *p* < 0.01). Excluding Michaëlsson et al. [6] from the analysis resulted in a SRR of 0.99 (95% CI: 0.95–1.03), with a somewhat reduced heterogeneity (I^2^ = 63%, *p* < 0.01) (Additional file 2: Table S2).

**Fig. 3:**
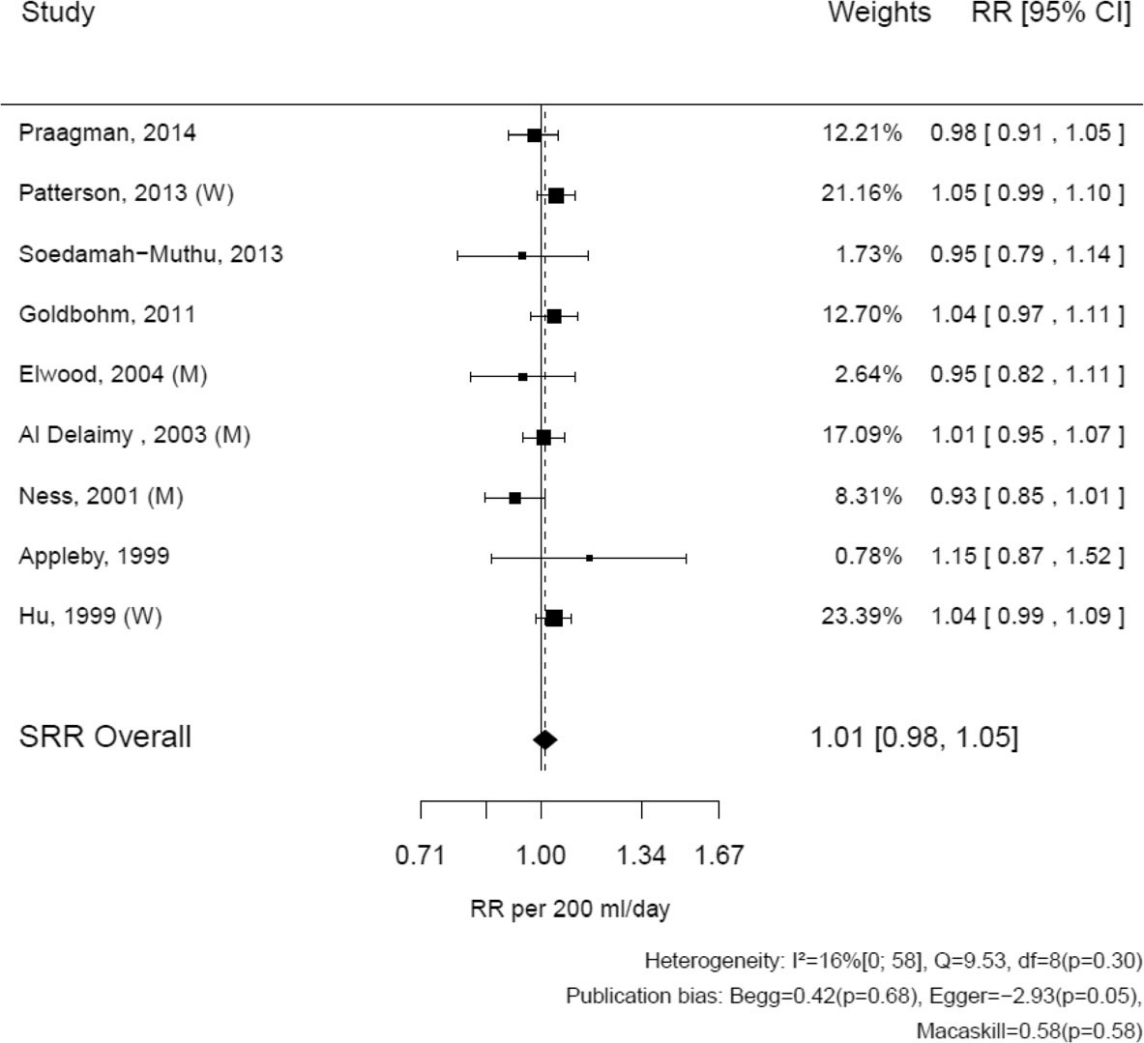
Relation between milk (per 200 mL/d) and fatal and non fatal coronary heart disease: dose-response meta-analyses of nine prospective cohorts. Author names, year of publication, and the size of the association per study; the *horizontal lines* indicate 95% confidence intervals. The last two columns contain the actual estimated relative risks and 95% confidence intervals. On the x axis, a line is plotted through the relative risk = 1. The diamond at the *bottom* indicates the summary relative risk with 95% confidence interval. Tests for heterogeneity and publication bias are mentioned at the *bottom*

For fatal and non-fatal coronary heart diseases, relative risks reported by studies ranged from 0.93 (95% CI: 0.85–1.01) to 1.15 (95% CI: 0.87–1.52) (Fig. 3) [10, 19]. The SRR of fatal and non-fatal coronary heart disease associated with the consumption of 200 mL/ of milk was 1.01 (95% CI: 0.98–1.05). The heterogeneity of results between studies was moderate (I^2^ = 16%, *p* = 0.30). Excluding Hu et al. [18] from the analysis did not alter the results (Additional file 2: Table S2).

**Fig. 4:**
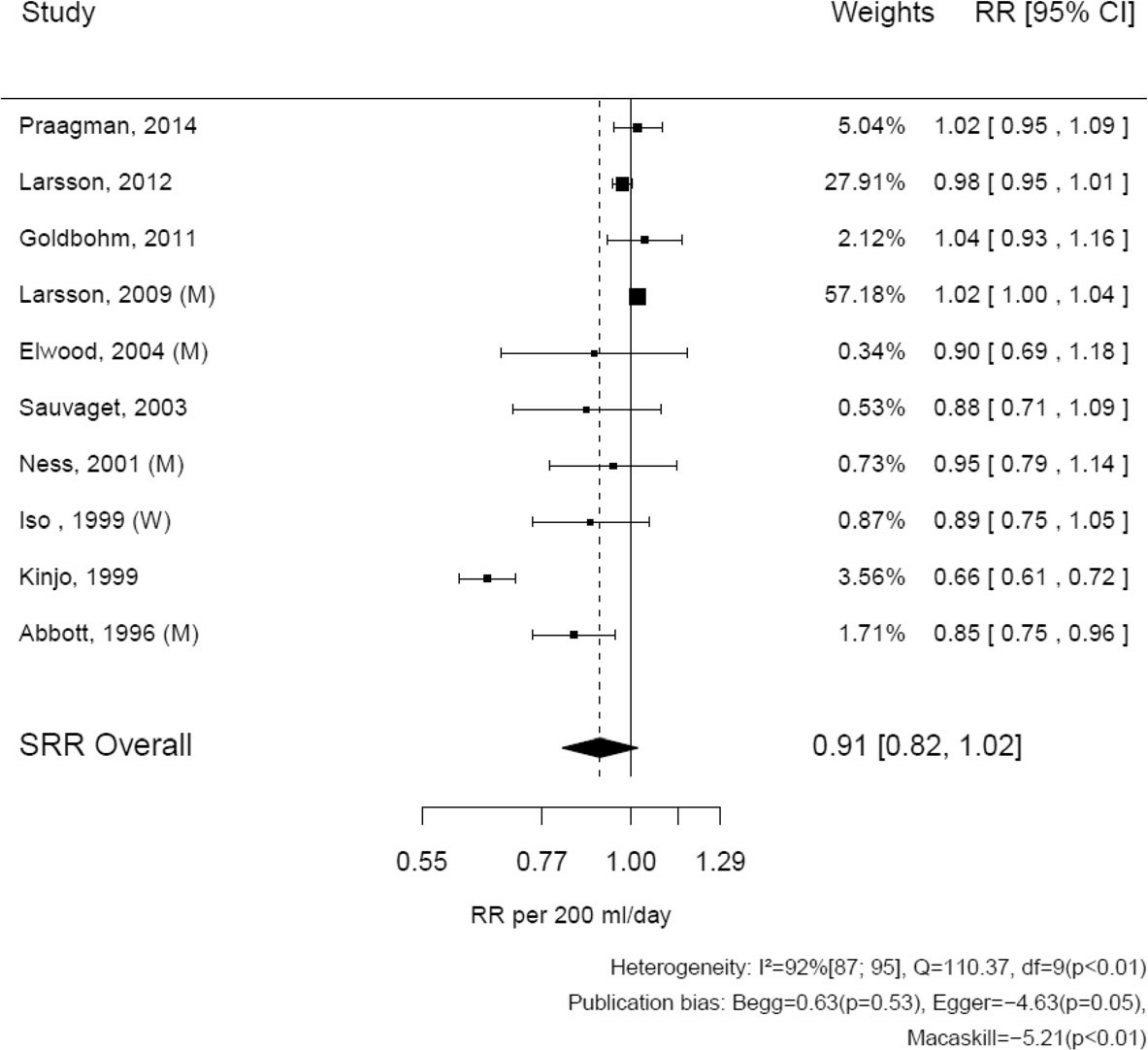
Relation between milk (per 200 mL/d) and fatal and non fatal stroke: dose-response meta-analyses of ten prospective cohorts. Author names, year of publication, and the size of the association per study; the *horizontal lines* indicate 95% confidence intervals. The last two columns contain the actual estimated relative risks and 95% confidence intervals. On the x axis, a line is plotted through the relative risk = 1. The diamond at the *bottom* indicates the summary relative risk with 95% confidence interval. Tests for heterogeneity and publication bias are mentioned at the *bottom*

For fatal and non-fatal stroke, relative risks reported by studies ranged from 0.66 (95%: 0.61–0.72) to 1.04 (95% CI: 0.93–1.16) (Fig. 4) [13, 29]. The SRR of fatal and non-fatal stroke associated with consumption of 200 mL/d of milk was 0.91 (95% CI: 0.82–1.02). The heterogeneity of results across studies was considerable (I^2^ = 92%, *p* < 0.01).

Stratified analyses restricted to men and adjustments for age, BMI, total energy intake and physical activity did not alter the estimates (Additional file 2: Table S2). The influences of individual studies with deviating results were tested in a sensitivity analysis. For all-cause mortality, exclusion of Michaëlsson et al. [6] did not influence the SRR, nor did the exclusion of Hu et al. [18] for fatal and non fatal coronary heart disease. Data from Abbott et al. [16] were extracted from a figure, but excluding the study did not influence the SRR.

No study reported results separately for skimmed and for whole milk, except three [13, 17, 18]. Hu et al. [18] compared the coronary heart disease risk of two or more glasses/d milk versus never. They found a relative risk of 1.67 (95%CI: 1.14–1.90) for whole milk, and of 0.78 (95%CI: 0.63–0.96) for skimmed milk. Goldbohm et al. [13] found no differences in mortality, ischemic heart disease and stroke between whole and skimmed milk. Larsson et al. [17] found no differences in risk of stroke between whole milk and low fat milk.

Despite the relatively small number of published data, the Macaskill test and to some extent, the Egger test suggested the presence of publication bias for all cause mortality and fatal and non-fatal stroke (Additional file 2: Table S2). Funnel plots are graphs in which the relative risks reported by studies are plotted against the inverse of relative risk variances. Relative risks of large size studies have smaller variance and are located in the upper part of the plots. Relative risks of small size studies tend to be more variable and have a greater variance. Small size studies are located in the lower parts of the plots. The Additional file 2: Figure S1 shows an assymetry in the spread of relative risks in the plot, with a gap in the right lower part of the plot. These gaps suggest that a number of smaller size studies that found increased risks of all cause mortality were not published. The Funnel plots for coronary heart disease and for stroke are less indicative of publication bias.

The main results of the two studies that could not be integrated in the meta-analysis were a relative risk of coronary heart disease of 0.99 (95% CI: 0.71–1.38) in men and of 1.01 (95% CI: 0.68–1.49) in women associated with sometimes/often whole milk consumption relative to never/rare milk consumption [42]. From a multivariate model, Snowdon et al. [43] reported relative risks of 0.94 (*p* < 0.05) in men and of 1.11 (*p* > 0.05) in women when comparing two glasses of milk a day versus none.

## Discussion

Our meta-analysis of observational prospective studies found no evidence for associations between milk consumption and all-cause mortality, fatal and non fatal coronary heart diseases and fatal or non fatal stroke. The possibility of publication bias is however possible that may have resulted in less published data suggesting increased risk of all-cause mortality, and perhaps also of stroke, associated with increasing milk consumption.

Our meta-analysis has several limitations. We could not integrate Avalos et al. [42] and Snowdon et al. [43] because a dichotomous variable was used for reporting results. We did not take cardiovascular diseases as outcome, because the definition of this condition was heterogeneous across studies. We also excluded two studies because data specific to milk consumption were not reported [36, 37]. For instance, the study of Fortes et al. [36], that was included in Soedamah-Muthu et al. [5], found an implausible relative risk of cardiovascular mortality of 0.24 (95% CI: 0.07–0.86), associated with consumption of milk and yoghurt together. Each study expressed dairy consumption using different units (pints, frequency/week, times/day, and servings/week), and assumptions about the volume of a serving had to be made to convert values into mL/d.

The contradictory conclusions reached by the meta-analysis of Soedamah-Muthu et al. [5] and by the Swedish study [6] may be regarded as a reflect of the considerable heterogeneity in results obtained by prospective studies on milk consumption and all-cause mortality, coronary heart disease and stroke. Several reasons may underlie the heterogeneity in results obtained by studies, like selection of subjects included in cohorts, the way milk consumption was assessed, the types of milk consumed, and the adjustments done. In most studies, a single measure of exposure was done at baseline, which can lead to misclassification of exposure during follow-up. Milk is never consumed alone, but is part of a global nutritional and lifestyle behaviour. After decennia of promotion by governements and by advertising campaigns, milk has acquired a “healthy” image for the public, and has become considered as being a natural component of a healthy diet. For example, four prospective studies [10, 13, 33, 44] found lower prevalences of smokers in milk drinkers, and two [13, 44] found higher physical activity and education in daily milk consumers. As a result, it can not be ruled out that high milk consumption would be part of a health conscious behavioural cluster, which will be difficult to correct in multivariate analysis. Michaëlsson et al. [6] found that intake of 200 mL/d of milk was associated with a relative risk for all-cause mortality of 1.15 (95% CI: 1.12–1.19) in women and 1.03 (95% CI: 1.01–1.04) in men. Such increased risk in women was never found in other studies. In Michaëlsson et al. [6], current smoking status increased with increasing milk consumption, when other propective studies found the reverse, that is lower smoking rates associated with greater milk consumption [10, 13, 33, 44]. It could well be that in the Swedish cohort, women who smoked tended to drink more milk because of healthy virtues, like the prevention of bone demineralisation. Osteoporotic fractures are two to three times more frequent in women than in men, and Sweden has one of the highest incidence of osteoporotic fractures in the world [45]. It is also predicted that within the next 40 years or so, the annual number of hip fractures in Sweden will double [46]. One could speculate that, considering the high public awareness about osteoporotic fractures in Sweden, high milk consumption would be part of a more general behaviour intended to prevent osteoporotic fractures. If women at higher risk of osteoporotic fracture because they smoke or are physically inactive also drink more milk, then high milk consumption could appear being associated with a higher risk of cardiovascular events. Multivariate analysis allows adjustment for potential confounders, but residual confounding may remain, due to inaccurate measurement. Lastly, even if measurements were perfect, multicollinearity is likely to threaten the correct interpretation of multivariate models.

The meta-analysis of Soedamah-Muthu et al. [5] found an inverse relationship between milk consumption and cardiovascular disease, based on four prospective studies. In three prospective studies, there was a heterogenous definition of cardiovascular diseases going from only mortality to coronary heart disease, stroke, cardiac arrest, heart failure and sudden death. The study by Engberink et al. [37], that was included in Soedamah-Muthu et al. [5], is a poster on the Rotterdam Study presented at the annual scientific meeting of the American Heart Association in 2010, in which no risk estimates for milk consumption and cardiovascular disease was reported. Results of the Rotterdam Study were published in a further publication by Praagman et al. [33], which we included in our meta-analysis. However, Praagman et al. [33] reported results for coronary heart disease and stroke, but not for cardiovascular disease.

## Conclusion

Our meta-analysis of observational prospective studies found no evidence of an association between milk consumption and all-cause mortality, fatal and non fatal coronary heart disease and fatal and non fatal stroke. However, the possibility cannot be dismissed that a publication bias could lead to an underestimation of risks associated with milk consumption.

## Additional files

Additional file 1: Table S1. Characteristics of prospective cohort studies on milk consumption and all-cause mortality, fatal and non-fatal coronary heart disease and stroke. (DOCX 145 kb)
Additional file 2: Table S2. Summary of the results of meta-analyses and sensitivity analyses for 200 mL/d milk and all cause mortality, fatal and non fatal coronary heart disease and stroke. (DOCX 134 kb)

## Abbreviations

BMI: Body mass index
CI: Confidence interval
ICD-10: International Classification of Diseases and Related Health Problems
SRR: Summary relative risk
