# Supplementary material for: Daily milk consumption and all-cause mortality, coronary heart disease and stroke: a systematic review and meta-analysis of observational cohort studies
Source: BMC Public Health. 2016 Dec 8;16:1236. doi: 10.1186/s12889-016-3889-9 (PMC5143456; doi:10.1186/s12889-016-3889-9)
Supplement: Additional file 2: Table S2. — Summary of the results of meta-analyses and sensitivity analyses for 200 mL/d milk and all cause mortality, fatal and non fatal coronary heart disease and stroke. (DOCX 134 kb) [file 12889_2016_3889_MOESM2_ESM.docx]

Table S2. Summary of the results of meta-analyses and sensitivity analyses for 200 mL/d milk and all cause mortality, fatal and non fatal coronary heart disease and stroke.

| Analyses | No. Cohorts | SRR | 95% CI | | I^2^ (%) | 95% CI | | Begg test | Egger test | Macaskill test |
| --- | --- | --- | --- | --- | --- | --- | --- | --- | --- | --- |
| **All cause mortality** |  |  |  |  |  |  |  |  |  |  |
| All cohorts | 11 | 1.01 | 0.96 | 1.06 | 94 | 92 | 96 | 0.76 | 0.64 | <0.01 |
| Men only | 4 | 1.00 | 0.92 | 1.09 | 81 | 50 | 93 | 1.00 | 0.24 | 0.38 |
| Adjusted for age, BMI, TEI, PA | 6 | 1.04 | 0.95 | 1.13 | 96 | 93 | 97 | 0.85 | 0.67 | <0.01 |
| All but Michaëlsson et al. | 9 | 0.99 | 0.95 | 1.03 | 63 | 24 | 82 | 0.83 | 0.15 | 0.24 |
| **Fatal and non fatal coronary heart disease** |  |  |  |  |  |  |  |  |  |  |
| All cohorts | 9 | 1.01 | 0.98 | 1.05 | 16 | 0 | 58 | 0.68 | 0.05 | 0.58 |
| Men only | 4 | 0.99 | 0.91 | 1.08 | 25 | 0 | 88 | 0.17 | 0.61 | 0.50 |
| Adjusted for age, BMI, TEI, PA | 4 | 1.03 | 0.97 | 1.08 | 0 | 0 | 65 | 0.50 | 0.58 | 0.98 |
| All but Hu et al. | 8 | 1.01 | 0.96 | 1.05 | 18 | 0 | 61 | 0.46 | 0.11 | 0.54 |
| **Fatal and non fatal stroke** |  |  |  |  |  |  |  |  |  |  |
| All cohorts | 10 | 0.91 | 0.82 | 1.02 | 92 | 87 | 95 | 0.53 | 0.05 | <0.01 |
| Men only | 5 | 0.96 | 0.86 | 1.09 | 60 | 0 | 85 | 0.62 | 0.24 | 0.03 |
| All but Abbott et al. | 9 | 0.92 | 0.82 | 1.04 | 92 | 88 | 95 | 0.60 | 0.07 | <0.01 |
|  |  |  |  |  |  |  |  |  |  |  |
| SRR = summary relative risk; CI = confidence interval; BMI = Body Mass Index; TEI = total energy intake; PA = physical activity | | | | | | | | | | |

**References**

1. Kahn HA, Phillips RL, Snowdon DA, Choi W. Association between reported diet and all-cause mortality. Twenty-one-year follow-up on 27,530 adult Seventh-Day Adventists. Am J Epidemiol 1984;119(5):775-87.

2. Mann JI, Appleby PN, Key TJ, Thorogood M. Dietary determinants of ischaemic heart disease in health conscious individuals. Heart 1997;78(5):450-5.

3. Ness AR, Smith GD, Hart C. Milk, coronary heart disease and mortality. J Epidemiol Community Health 2001;55(6):379-82.

4. Elwood PC, Pickering JE, Fehily AM, Hughes J, Ness AR. Milk drinking, ischaemic heart disease and ischaemic stroke I. Evidence from the Caerphilly cohort. Eur J Clin Nutr 2004;58(5):711-7.

5. Paganini-Hill A, Kawas CH, Corrada MM. Non-alcoholic beverage and caffeine consumption and mortality: the Leisure World Cohort Study. Prev Med 2007;44(4):305-10.

6. Bonthuis M, Hughes MC, Ibiebele TI, Green AC, van der Pols JC. Dairy consumption and patterns of mortality of Australian adults. Eur J Clin Nutr 2010;64(6):569-77.

7. Goldbohm RA, Chorus AM, Galindo Garre F, Schouten LJ, van den Brandt PA. Dairy consumption and 10-y total and cardiovascular mortality: a prospective cohort study in the Netherlands. Am J Clin Nutr 2011;93(3):615-27.

8. van Aerde MA, Soedamah-Muthu SS, Geleijnse JM, Snijder MB, Nijpels G, Stehouwer CD, et al. Dairy intake in relation to cardiovascular disease mortality and all-cause mortality: the Hoorn Study. Eur J Nutr 2013;52(2):609-16.

9. Soedamah-Muthu SS, Masset G, Verberne L, Geleijnse JM, Brunner EJ. Consumption of dairy products and associations with incident diabetes, CHD and mortality in the Whitehall II study. Br J Nutr 2013;109(4):718-26.

10. Michaelsson K, Wolk A, Langenskiold S, Basu S, Warensjo Lemming E, Melhus H, et al. Milk intake and risk of mortality and fractures in women and men: cohort studies. Bmj 2014;349:g6015.

11. Hu FB, Stampfer MJ, Manson JE, Ascherio A, Colditz G, Speizer FE, et al. Dietary saturated fats and their food sources in relation to the risk of coronary heart disease in women. Am J Clin Nutr 1999;70:1001-8.

12. Appleby PN, Thorogood M, Mann JI, Key TJ. The Oxford Vegetarian Study: an overview. Am J Clin Nutr 1999;70(3 Suppl):525S-531S.

13. Al-Delaimy WK, Rimm EB, Willett W, Stampfer M, Hu F. A prospective study of calcium intake from diet and supplements and risk of ischemic heart disease among men. Am J Clin Nutr 2003;77:814-818.

14. Patterson E, Larsson SC, Wolk A, Akesson A. Association between dairy food consumption and risk of myocardial infarction in women differs by type of dairy food. J Nutr 2013;143(1):74-9.

15. Praagman J, Franco OH, Ikram MA, Soedamah-Muthu SS, Engberink MF, van Rooij FJ, et al. Dairy products and the risk of stroke and coronary heart disease: the Rotterdam Study. Eur J Nutr 2015;54(6):981-90.

16. Abbott RD, Curb JD, Rodriguez BL, Sharp DS, Burchfiel CM, Yano K. Effect of dietary calcium and milk consumption on risk of thromboembolic stroke in older middle-aged men. The Honolulu Heart Program. Stroke 1996;27(5):813-8.

17. Iso H, Stampfer MJ, Manson JE, Rexrode K, Hennekens CH, Colditz GA, et al. Prospective Study of Calcium, Potassium, and Magnesium Intake and Risk of Stroke in Women. Stroke 1999;30(9):1772-1779.

18. Kinjo Y, Beral V, Akiba S, Key T, Mizuno S, Appleby P, et al. Possible protective effect of milk, meat and fish for cerobrovascular disease mortality in Japan. J Epidemiol 1999;9(4):268-74.

19. Sauvaget C, Nagano J, Allen N, Grant EJ, Beral V. Intake of animal products and stroke mortality in the Hiroshima/Nagasaki Life Span Study. Int J Epidemiol 2003;32(4):536-43.

20. Larsson SC, Mannisto S, Virtanen MJ, Kontto J, Albanes D, Virtamo J. Dairy foods and risk of stroke. Epidemiology 2009;20(3):355-60.

21. Larsson SC, Virtamo J, Wolk A. Dairy consumption and risk of stroke in Swedish women and men. Stroke 2012;43(7):1775-80.

Figure S1. Funnel plot of relative risks reported by observational studies on milk and all-cause mortality according to the study size as estimated by the inverse of the relative risk variance. Relative risks are for a consumption of per 200 mL/d.

Figure S2. Funnel plot of relative risks reported by observational studies on milk and fatal and non-fatal coronary heart disease according to the study size as estimated by the inverse of the relative risk variance. Relative risks are for a consumption of per 200 mL/d.

Figure S3. Funnel plot of relative risks reported by observational studies on milk and fatal and non-fatal stroke according to the study size as estimated by the inverse of the relative risk variance. Relative risks are for a consumption of per 200 mL/d.
